# Tunable broadband polarization converters based on coded graphene metasurfaces

**DOI:** 10.1038/s41598-020-80493-w

**Published:** 2021-01-14

**Authors:** Ali Khajeh, Zahra Hamzavi-Zarghani, Alireza Yahaghi, Ali Farmani

**Affiliations:** 1 School of Electrical and Computer Engineering, Shiraz University, Shiraz, Iran; 2 Dipartimento di Elettronica e Telecomunicazioni, Politecnico di Torino, Turin, Italy; 3 Optoelectronic Research Center, Department of Electrical and Computer Engineering, Lorestan University, Khorramabad, Iran

**Keywords:** Optical materials and structures, Optical techniques

## Abstract

In this paper, two optimization algorithms (randomly initialized hill climbing and genetic algorithms) are considered to design broadband polarization converters based on coded metasurfaces. A pixeled graphene patch with an elliptic structure is proposed for the initial solution. Each pixel can be 1 and 0 which represents the presence and absence of the graphene. The initial guess tends to the optimum configuration after several optimization processes. Four broadband polarization converters are designed utilizing the optimization algorithms. By changing the chemical potential of graphene, the operation frequency of the polarization converters can be adjusted. Furthermore, the effects of relaxation time of graphene and incident angle on the polarization conversion bandwidth of the four designed structures are investigated.

## Introduction

Polarization state of electromagnetic waves can be manipulated by polarization converters which are useful in several applications such as optical communication^1^, imaging^2^ and detection^3^. Since, polarization state is an important feature of the waves, many researches have been done to design polarization converters based on optical gratings, dichroic crystals and birefringence effect^4^. These conventional methods are realized by bulky structures because long distance is needed for phase accumulation. Recently, a lot of investigations have been carried out to design compact and low profile polarization converters for integration and miniaturization purposes^5–7^. Metasurfaces which are 2-D arrays of scattering particles are good candidates for design of thin polarization converters^8^.

To obtain tunability, graphene which is a tunable material with respect to the frequency and applied bias voltage is utilized. It has extraordinary electrical, optical and mechanical properties^9^. Since graphene supports the propagation of surface plasmon polaritons at terahertz and infrared frequencies, graphene is applied in tunable and reconfigurable devices such as absorbers^10,11^, modulators^12^, detectors^13^, switches^14^ and lenses^15^.

The tunability of graphene based metasurfaces is obtained by adjusting the chemical potential of graphene with different applied bias voltages^16^. In recent years a lot of graphene based patterned metasurfaces have been presented as polarization converters for terahertz and midinfrared frequencies^17,18^.

In this paper, four coded metasurfaces to rotate the linear polarization of the reflected wave are proposed relying on randomly initialized hill climbing and genetic optimization algorithms. The metasurfaces are constructed of digital units. Broadband polarization converters based on coded metasurfaces are achieved with our proposed method. To the best of our knowledge, no research has been reported on the tunable broadband polarization converter based on coded metasurfaces. The frequency range is considered between 20 and 40 THz for our four structures and the periodicity in both *x* and *y* directions is fixed to 200 nm.

Randomly initialized hill climbing is one of the most basic methods among optimization algorithms. This method starts with a random initial solution and then all neighbors of the initial answer are examined by the objective function. The neighbor with the best answer replaces the initial point and the algorithm will continue until no more acceptable neighbor is found. Here, the objective function is the bandwidth of the polarization conversion ratio and the neighbor with the largest bandwidth is chosen^19^.

The genetic algorithm is inspired by genetic science and Darwin theory of evolution. This optimization algorithm is a population based method and it is capable to solve both continuous and discontinuous problems. The genetic algorithm is a directional random optimization method which tends gradually to the optimum point. It starts with a set of solutions named the initial population. Each member of this population is named chromosome. In each generation, the chromosomes are examined and according to their values can survive and multiply. The examination of chromosomes is done using a fitness function. Generation in the genetic algorithm is done utilizing crossover and mutation functions. The chromosomes become more perfect during several generations and it continues until the stop condition is obtained^20^.

The paper is structured as follows: “Design procedure” reports the design and optimization of the polarization converter unit cell. In “Result and discussion”, the optimum structures which are optimized by randomly initialized hill climbing and genetic algorithms are presented and results of PCR are shown. The effect of tunability and relaxation time of graphene and incident angle is also studied. In next section, some conclusions are drawn.

## Design procedure

Design of an optimal broadband polarization converter based on metasurfaces includes determination of periodicity of the unit cell, substrate height and dielectric constant and patch arrangement. In two structures SiO$_2$ with a dielectric constant of 3.9 and in the other two structures Polymethylpentene (TPX) with a dielectric constant of 2.1 are utilized as the substrate which produce low loss in the considered range of frequency. The substrate is grounded by a gold layer with a conductivity of $4.56\times 10^7$ s/m and thickness of 100 nm. The metasurface is placed on top of the substrate which should be optimized to achieve a broadband performance for the polarization converter. Genetic and randomly initialized hill climbing (RHC) optimization algorithms are assumed for this purpose. The unit cell of the metasurfaces is divided into $10\times 10$ pixels. The optimization is applied to the pixels which are coded with a binary code. Each pixel can be 1 or 0 representing the presence or absence of graphene. As mentioned before, graphene is used in the structure of the designed metasurface for tunability performance. Its conductivity is adjustable by changing frequency and the chemical potential of graphene with different bias voltages which can be applied to graphene patches by adding a gel ionic layer on top of them^21,22^. Kubo formula which models the graphene conductivity shows its dependence to the frequency and the chemical potential as follows^23^:

1 $$\begin{aligned} \sigma _{intra}= & {} -j \frac{K_B e^2 T}{\pi \hbar ^2 (w-2j \tau ^{-1})} \left( \frac{\mu _c}{K_B T}+ 2 \ln \left( e^{-\frac{\mu _c}{K_B T}}+1\right) \right) \end{aligned}$$

2 $$\begin{aligned} \sigma _{inter}= & {} \frac{j e^2}{4 \pi \hbar } \ln \left( \frac{2 \vert \mu _c \vert -(w-j \tau ^{-1}) \hbar }{2 \vert \mu _c \vert +(w-j \tau ^{-1}) \hbar } \right) \end{aligned}$$

We used Eqs. (1) and (2) to obtain the conductivity of graphene. Then we modeled graphene as a surface with the obtained conductivity. We wrote a VBA code in Matlab which models the whole structure with boundary conditions. The VBA code was imported to CST to simulate the initial structure. The results imported to Matlab to optimize the structure. After finding the optimum unit cell by using optimization algorithms, it imports to CST and we can plot the optimum results.

For polarization rotation, the structure should be in 45$^\circ$ with respect to the polarization of the incident wave. Therefore, we rotate the pixeled structure 45$^\circ$ in the *x*–*y* plane. We also applied symmetry in the unit cell configuration. Therefore, by determination of 1/4 of the unit cell structure, the complete element will be defined.

**Figure 1:**
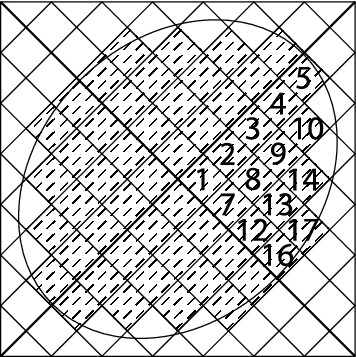
Pixeled metasurface inclined by an elliptic.

The initial structure of the metasurface is considered as an elliptic with $100 \times 80$ $nm^2$. The considered pixeled structure which is coded with a binary code of 111110111101110110000 is shown in Fig. 1.

Each produced binary code by the optimization algorithms is translated to the structure in CST Microwave Studio environment and the configuration corresponding to the binary code is simulated. The amplitude of the reflection coefficients for the two reflected waves polarized in *x* and *y* directions is obtained as the output. Polarization conversion ratio versus frequency can be calculated with these outputs from CST. The bandwidth as a fitness function is obtained in Matlab and the optimization is carried on based on the considered algorithms.

## Result and discussion

### Optimization with randomly initialized hill climbing

#### The first structure

In the first structure, optimization with the randomly initialized hill climbing algorithm is done with consideration of a substrate with a dielectric constant of 2.1 and the chemical potential of 0.8 eV. The structure corresponds to the binary code 001111110011100111111 represents the best result which means the largest bandwidth.

**Figure 2:**
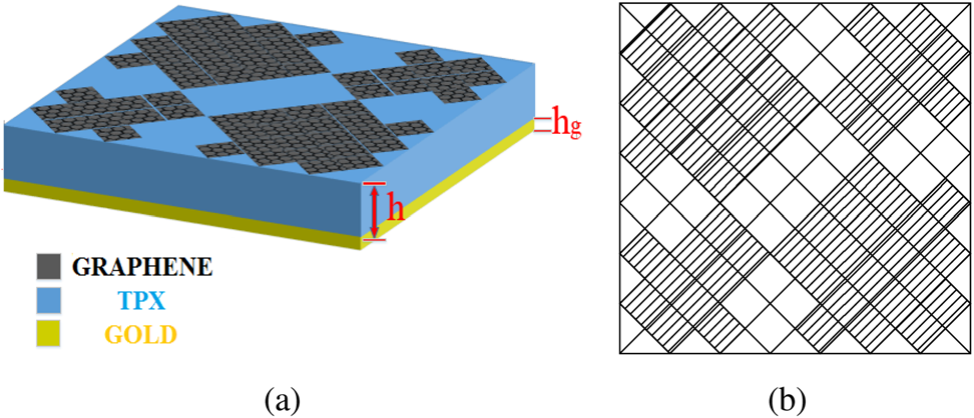
Structure of the fist optimized metasurface (**a**) 3-D view, (**b**) top view [001111110011100111111].

**Figure 3:**
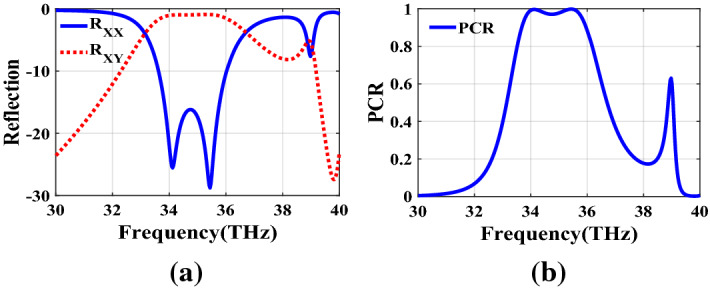
(**a**) Reflection coefficients of co and cross polarized reflected waves for the first structure, (**b**) PCR of the first structure.

**Figure 4:**
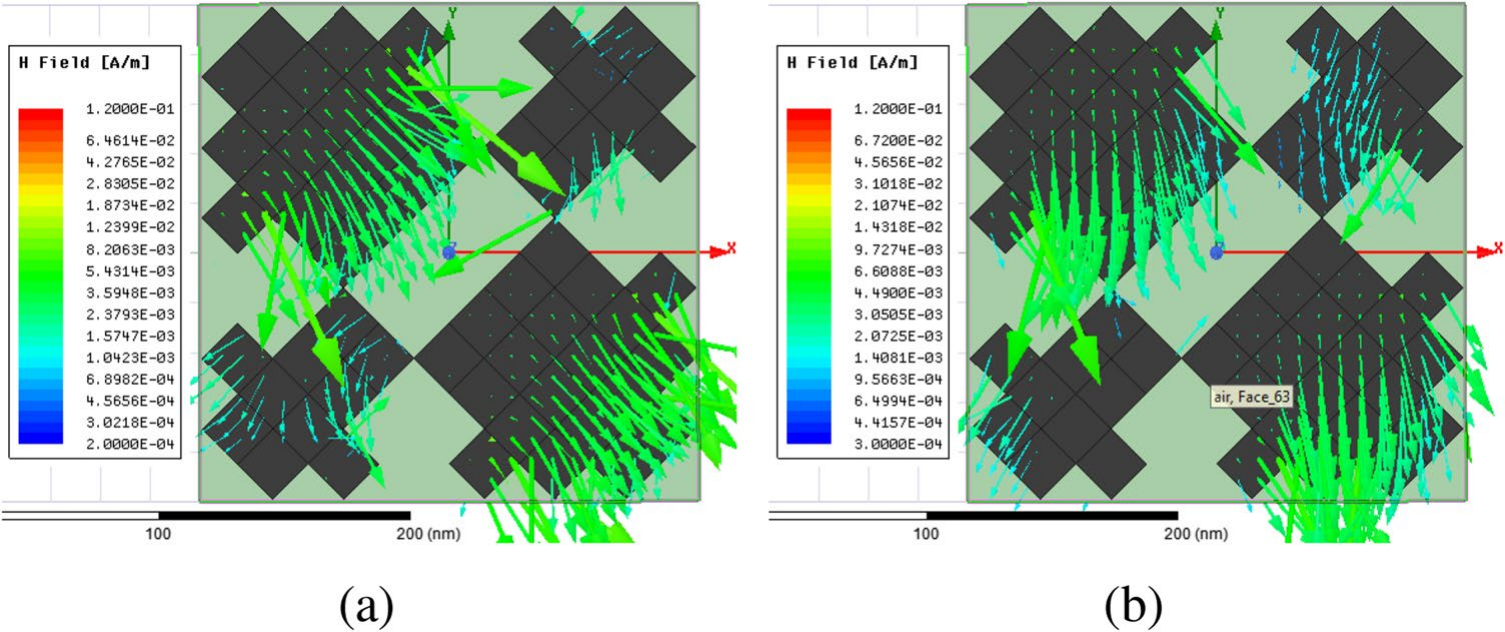
Magnetic field on the surface of the first structure (**a**) at 34.12 THz and (**b**) at 35.44. THz.

**Figure 5:**
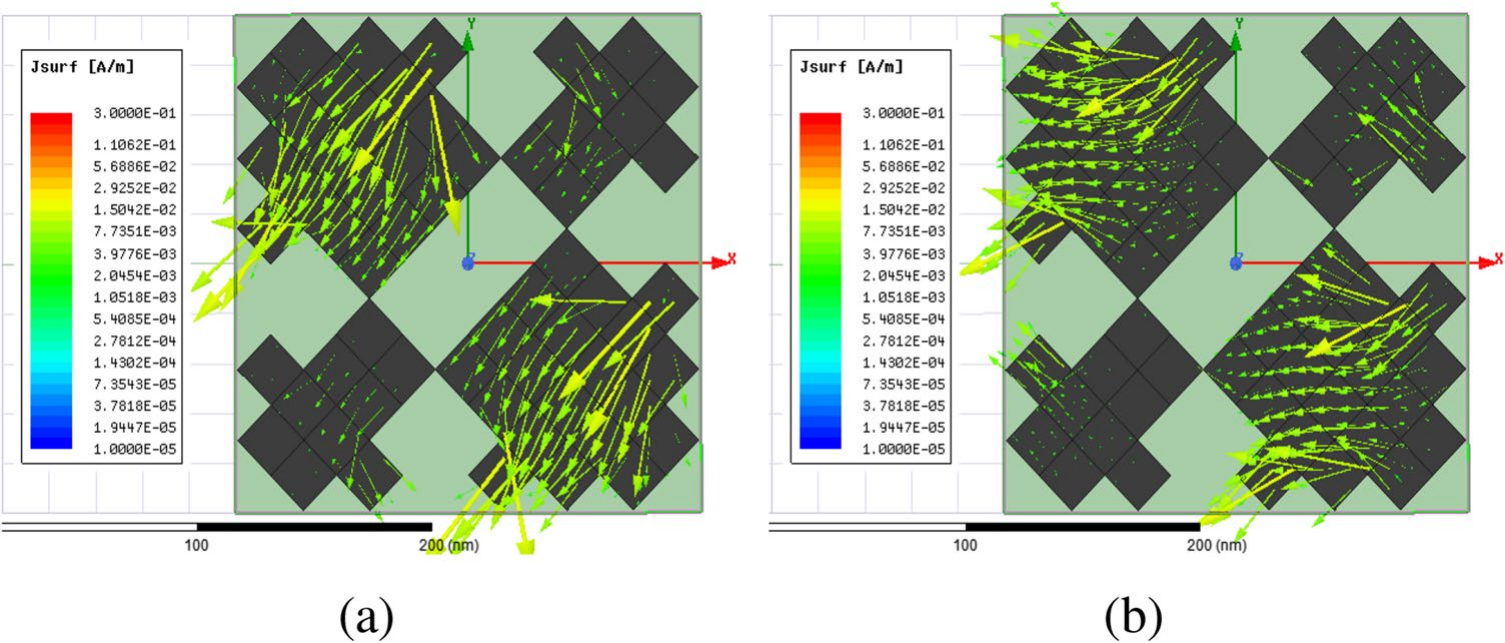
Surface current on the surface of the first structure at (**a**) 34.12 THz and (**b**) 35.44 THz.

**Figure 6:**
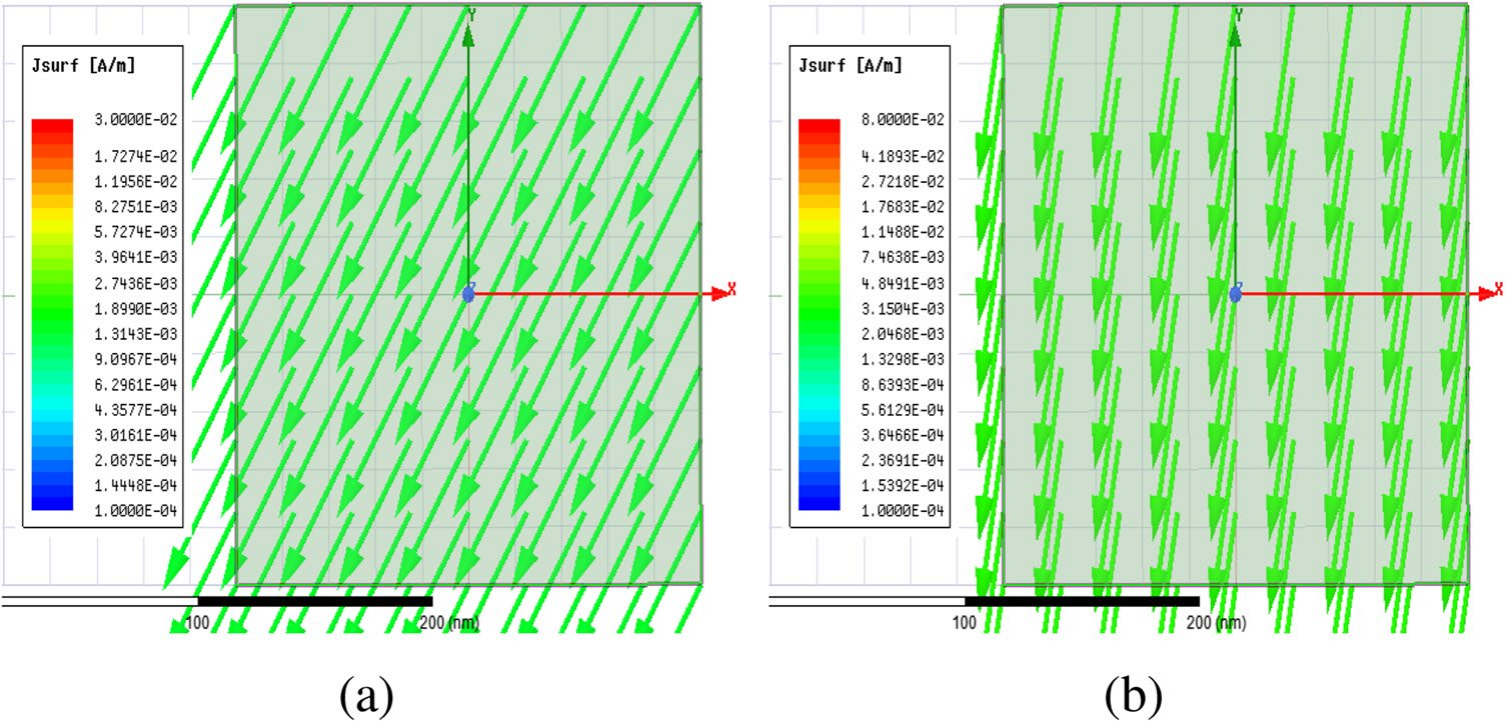
Surface current on the ground plane (**a**) at 34.12 THz and (**b**) at 35.44 THz.

**Figure 7:**
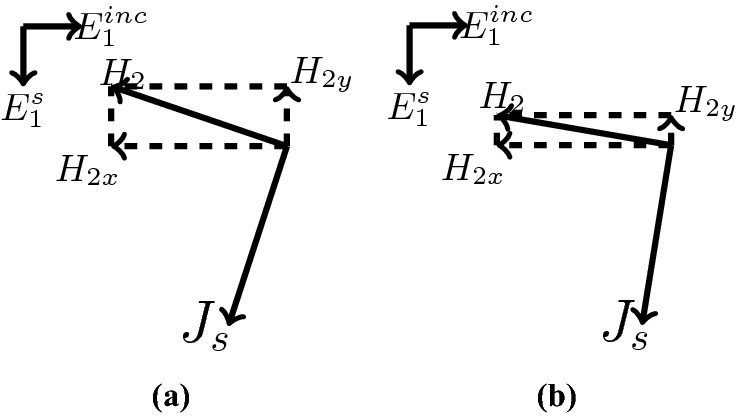
Induced current J on the ground plane.

Figure 2 shows the structure of the metasurface regarding the obtained binary code. The reflection coefficients of $R_{xx}$ and $R_{xy}$ representing co and cross-polarized reflection amplitude for the *x* polarized incident wave are shown in Fig. 3a. It can be seen two resonance frequencies at 34.12 THz and 35.44 THz. The magnetic fields and surface currents on the surface of the structure are shown in Figs. 4 and 5, respectively. These vectors are mostly in cross polarized direction. Figure 6 illustrates the induced current on the ground surface. According to Fig. 7, the induced current J on the ground plane produces the magnetic field $H_2$ which comprises *x* and *y* components. $H_{2y}$ relates to an electric field in the same direction as the incident wave while $H_{2x}$ relates to an electric field normal to the electric field of the incident wave which indicates the polarization rotation of the reflected wave. Polarization conversion ratio (PCR) is calculated from the following formula^24^ and it is plotted versus frequency in Fig. 3b.

3 $$\begin{aligned} PCR = \frac{|{R_{xy}}^2|}{|{R_{xx}}^2|+|{R_{xy}}^2|} \end{aligned}$$

**Figure 8:**
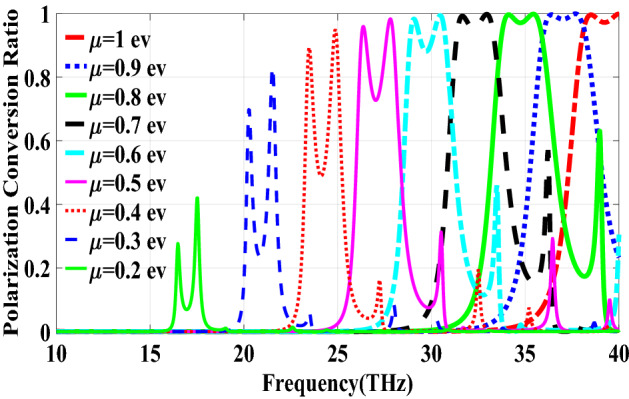
PCR of the first structure for different chemical potentials of graphene.

This polarization converter shows PCR of higher than 0.9 in the frequency range of 33.67–35.98 THz with 6.5 $\%$ bandwidth. Figure 8 shows the PCR of the structure for different chemical potentials of graphene. It indicates that by changing the chemical potential, the operation frequency range of the polarization converter can be adjusted. Furthermore, this structure can also be used as a switch. Indeed, for the values more than 0.6 eV it operates as a polarization converter with a tunable range of frequency but for the values smaller than 0.6 eV it does not work as a polarization converter anymore.

**Figure 9:**
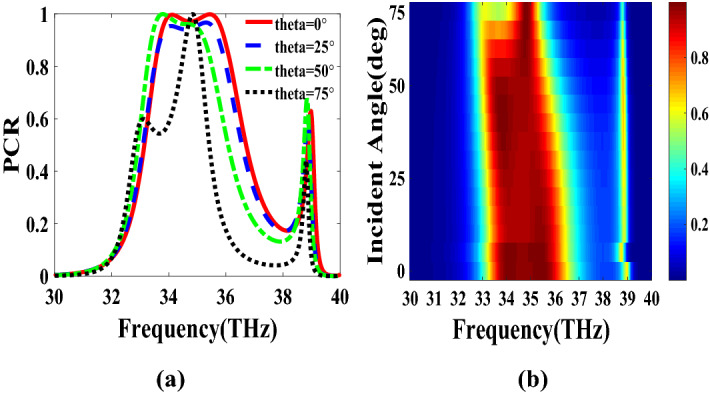
PCR of the first structure for different incident angles.

**Figure 10:**
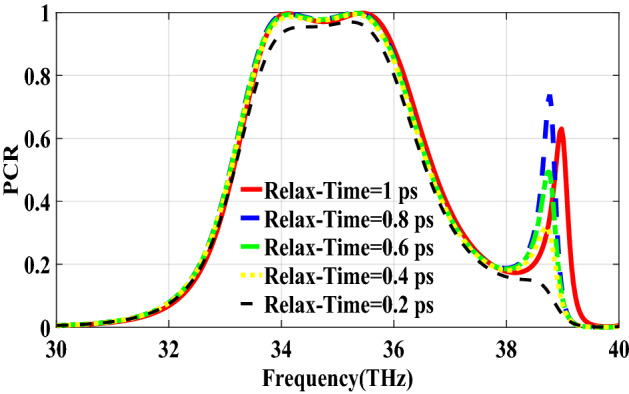
PCR of the first structure for different relaxation times of graphene.

PCR for different incident angles is also investigated and is shown in Fig. 9. It indicates that by increasing the incident angle, the PCR and bandwidth decrease and for the incident angles more than 60$^\circ$ the bandwidth is very small. Figure 10 shows the effect of the relaxation time of graphene on the operation of the designed polarization converter. It can be seen that the change of relaxation time does not have much have effect on the polarization conversion operation except that for the relaxation time of 0.2 eV the PCR and the bandwidth decrease slightly.

#### The second structure

**Figure 11:**
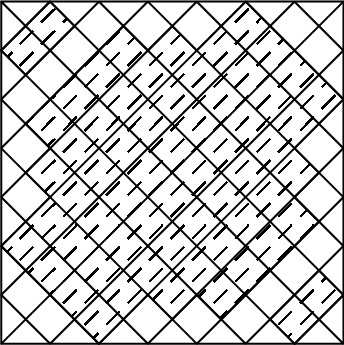
Structure of the second optimized metasurface [111110111111110110001].

**Figure 12:**
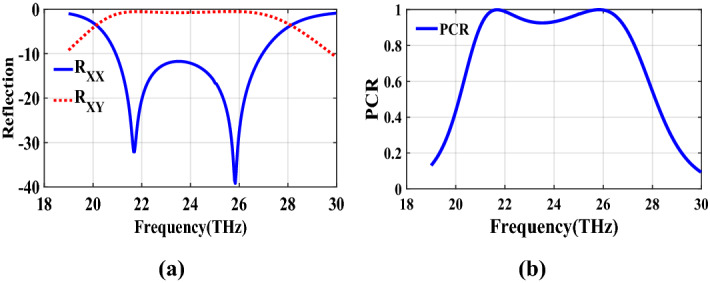
(**a**) Reflection coefficients of co and cross polarized reflected waves $R_{xx}$ and $R_{xy}$, (**b**) PCR of the second structure.

**Figure 13:**
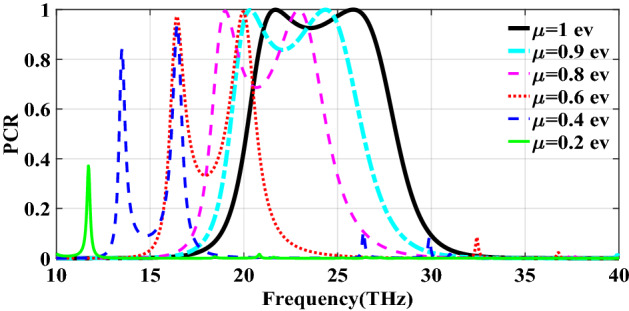
PCR of the second structure for different chemical potentials of graphene.

**Figure 14:**
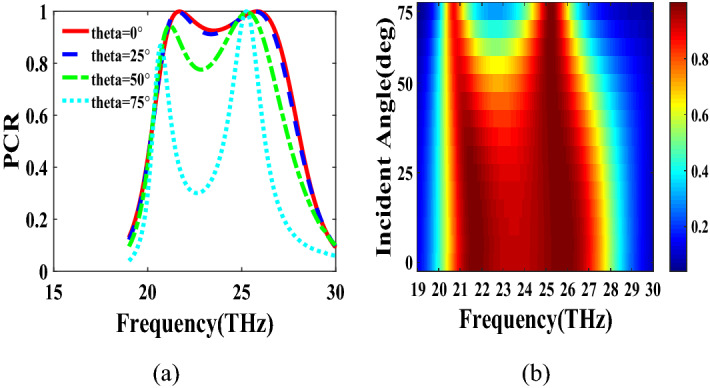
PCR of the second structure for different incident angles.

**Figure 15:**
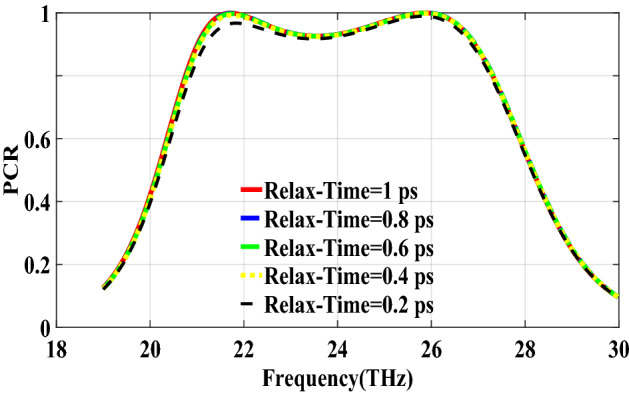
PCR of the second structure for different relaxation time of graphene.

The second structure which is optimized by randomly initialized hill climbing algorithm is considered with a substrate with a dielectric constant of 3.9 and the chemical potential of 1 eV. The binary code for the optimized metasurface which is shown in Fig. 11 is 111110111111110110001. The co and cross polarized reflection coefficients $R_{xx}$ and $R_{xy}$ are shown in Fig. 12a which indicated two resonance frequencies at 21.71 THz and 25.84 THz. The PCR for the reflection mode shown in Fig. 12b illustrates PCR more than 0.9 in the frequency range of 21–26.89 THz with a bandwidth of 24.6$\%$. The bandwidth of this structure is almost 20$\%$ more than of the first proposed. The tunability purposes of the designed polarization converter is proved in Fig. 13 by changing the chemical potential of graphene. Figure 14a shows PCR of the second structure corresponding to four different incident angles 0$^\circ$, 25$^\circ$, 50$^\circ$ and 75$^\circ$. Figure14b indicates that by increasing the incident angle, the performance of the polarization converter is degraded in both the ratio of the polarization conversion and the bandwidth. Figure 15 illustrates that changing the relaxation time of graphene has a very small impact on PCR of the designed second metasurface.

### Optimization with genetic algorithm

#### The third structure

**Figure 16:**
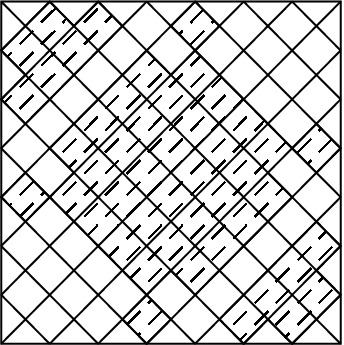
Structure of the second optimized metasurface 111000111001101000111.

**Figure 17:**
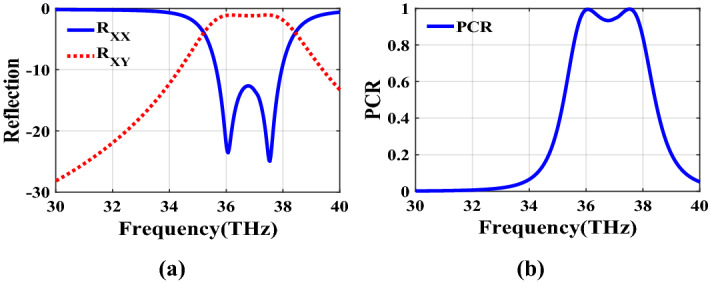
(**a**) Reflection coefficients of co and cross polarized reflected waves of the third structure, (**b**) PCR of the third structure.

**Figure 18:**
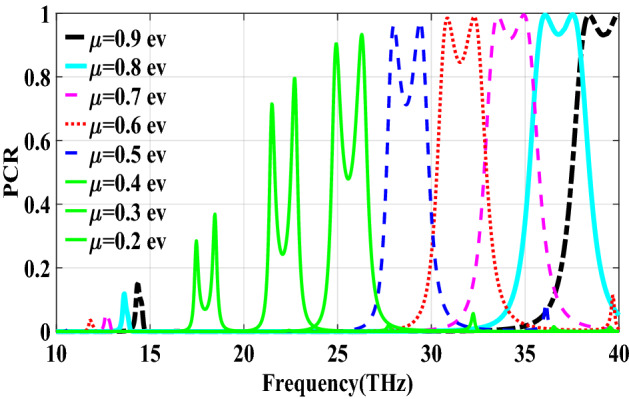
PCR of the third structure for different chemical potentials of graphene.

**Figure 19:**
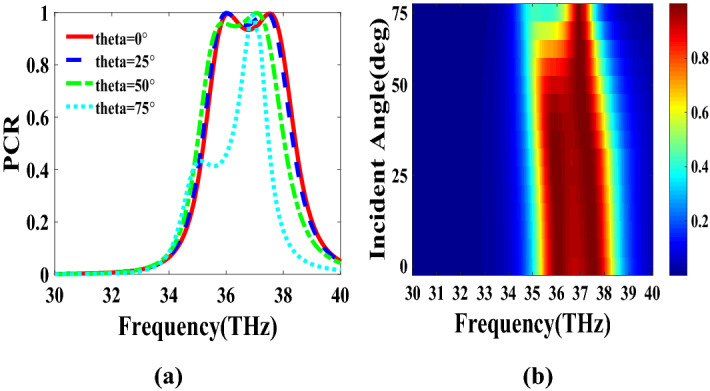
PCR of the third structure for different incident angles.

**Figure 20:**
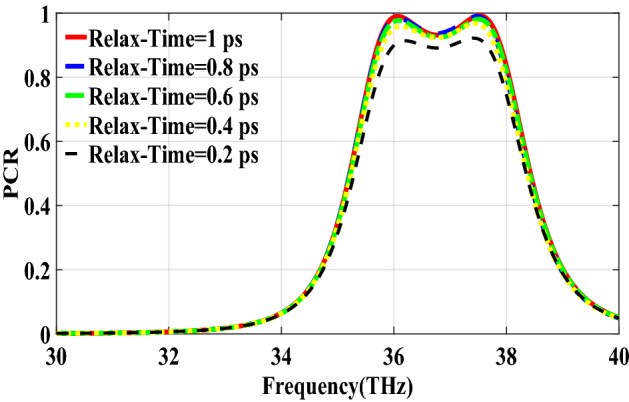
PCR of the third structure for different relaxation times of graphene.

In this section, two structures are optimized by the genetic algorithm to obtain the largest bandwidth. In the first structure, a substrate with a dielectric constant of 2.1 and the chemical potential of 0.8 eV is considered. Binary code of 111000111001101000111 is obtained for the optimum solution which we investigate in the following. The optimum structure for the third structure is shown in Fig. 16. The co and cross polarized reflection responses for the *x* polarized incident wave are illustrated in Fig. 17a indicating two resonance frequencies at 36.07 THz and 37.54 THz. According to co and cross polarized reflection coefficients, PCR is calculated and plotted versus frequency in Fig. 17b. The tunability property of graphene on the polarization conversion operation is shown in Fig. 18 which indicates that by increasing the chemical potential of graphene, the frequencies of high polarization conversion ratio increase. Moreover, the designed metasurface can work as a switch. Indeed, for chemical potential less than 0.5 eV it does not rotate the polarization of the reflected wave. Figure 19 shows PCRs for some incident angles. It can be seen that for the incident angles more than 60$^\circ$ the bandwidth of the PCR becomes very small. Figure 20 shows the impact of the relaxation time of graphene. For the relaxation time of 0.4, 0.6, 0.8 and 1 eV the result of PCR is almost the same but for the value of 0.2 eV, the level of PCR shrinks by 0.1.

#### The fourth structure

**Figure 21:**
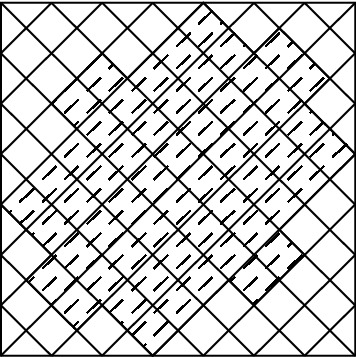
Structure of the fourth optimized metasurface 111110111101111100000.

**Figure 22:**
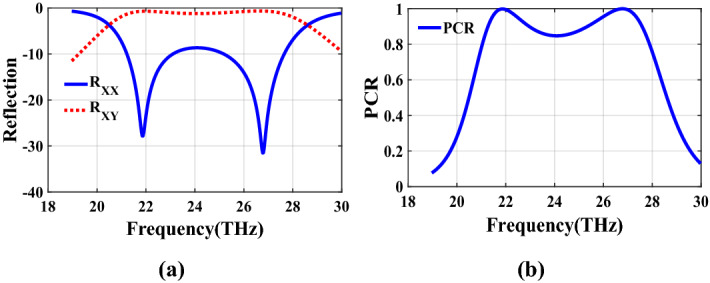
(**a**) Reflection coefficients of co and cross polarized reflected waves of the fourth structure, (**b**) PCR of the fourth structure.

**Figure 23:**
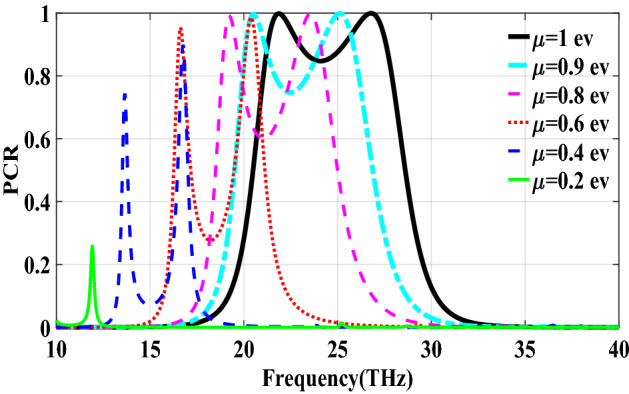
PCR of the fourth structure for different chemical potentials of graphene.

**Figure 24:**
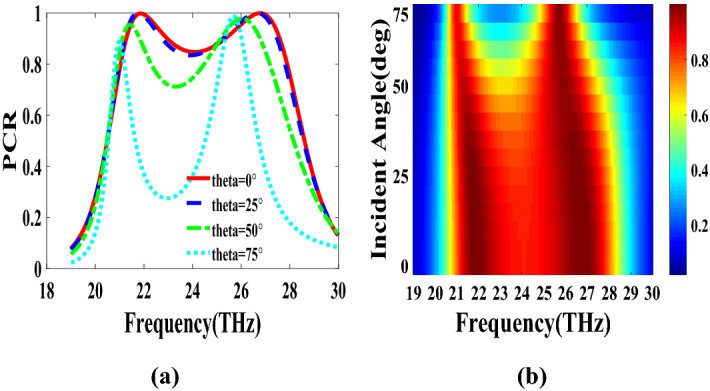
PCR of the fourth structure for different incident angles.

**Figure 25:**
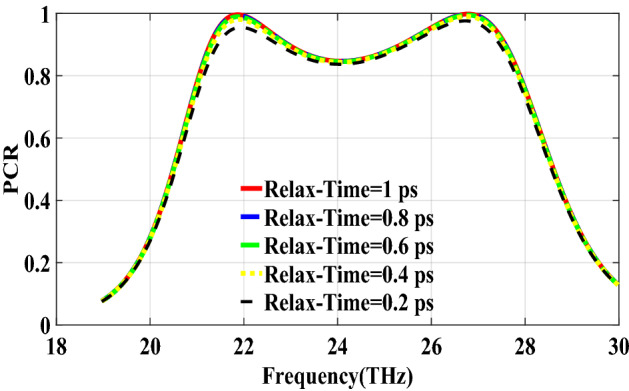
PCR of the fourth structure for different relaxation times of graphene.

The last structure is also optimized by the genetic algorithm. A substrate with a dielectric constant of 3.9 and a graphene layer with the chemical potential of 1 eV are used for this optimization process. The binary code of 111110111101111100000 is obtained after several generations. The optimized metasurface corresponding to this binary code is shown in Fig. 21. Figure 22a shows the amplitude of the co and cross polarized reflected waves. It can be seen two resonance frequencies at 21.88 THz and 26.8 THz. PCR of the fourth optimized structure is illustrated in Fig. 22b with a 25$\%$ bandwidth. Figure 23 shows the tunable performance of the polarization converter by changing the chemical potential of graphene. The effect of incident angle on the PCR is shown in Fig. 24 which indicates less bandwidth can be seen for the incident angles more than 60$^\circ$. Figure 25 proves that the relaxation time of graphene has a very small effect on PCR of the fourth polarization converter.

## Conclusion

In this paper, four polarization converters based on graphene pixeled metasurfaces have been designed. The structures have been optimized by randomly initialized hill climbing and genetic algorithms to obtain broad bandwidth polarization conversion. Graphene patches have been used in the configuration of the metasurface to achieve tunable performance. The first structure has a good performance in the frequency range of 34.2–35.44 THz with a chemical potential of 0.8 eV. The second structure operates properly in the frequency range of 21–26.89 THz with a chemical potential of 1 eV. The third structure works at the frequency range of 35.71–37.9 THz with a chemical potential of 0.8 eV and the fourth structure works at the frequency range of 21.28–27.58 THz with a chemical potential of 1 eV. All converters are tunable by changing their chemical potentials and perform well up to $60^\circ$ incident angle. However their operation bandwidth decrease with increasing the incident angle. The results also show that the value of relaxation time has a very small impact on the PCR of the structures.

## References

[1] Lajunen H, Turunen J, Tervo J (2005). Design of polarization gratings for broadband illumination. Opt. Express.

[2] Gruev V, Perkins R, York T (2010). CCD polarization imaging sensor with aluminum nanowire optical filters. Opt. Express.

[3] Zhao X, Boussaid F, Bermak A, Chigrinov V (2011). High-resolution thin “guest-host” micropolarizer arrays for visible imaging polarimetry. Opt. Express.

[4] Heismann F, Aferness R (1988). Wavelength-tunable electrooptic polarization conversion in birefringent waveguides. IEEE J. Quantum Electron..

[5] Yuan Y, Zhang K, Ratni B, Song Q, Ding X, Wu Q, Burokur Sh, Genevet P (2020). Independent phase modulation for quadruplex polarization channels enabled by chirality-assisted geometric-phase metasurfaces. Nat. Commun..

[6] Zhang K, Yuan Y, Ding X, Ratni B, Burokur Sh, Wu Q (2019). High efficiency metalenses with switchable functionalities in microwave region. ACS Appl. Mater. Interfaces.

[7] Yuan Y (2020). A fully phase-modulated metasurface as an energy-controllable circular polarization router. Adv. Sci..

[8] Perez-Palomino G, Page JE, Arrebola M, Encinar JA (2018). A design technique based on equivalent circuit and coupler theory for broadband linear to circular polarization converters in reflection or transmission mode. IEEE Trans. Antennas Propag..

[9] Vakil A, Engheta N (2011). Transformation optics using graphene. Science.

[10] Patel SK (2019). Graphene-based highly efficient and broadband solar absorber. Opt. Mater..

[11] Bordbar A, Basiry R, Yahaghi A (2020). Design and equivalent circuit model extraction of a broadband graphene metasurface absorber based on a hexagonal spider web structure in the terahertz band. Appl. Opt..

[12] Liu M (2011). A graphene-based broadband optical modulator. Nature.

[13] Gan X (2013). Chip-integrated ultrafast graphene photodetector with high responsivity. Nat. Photon..

[14] Farmani A, Yavarian M, Alighanbari A, Miri M, Sheikhi MH (2017). Tunable graphene plasmonic y-branch switch in the terahertz region using hexagonal boron nitride with electric and magnetic biasing. Appl. Opt..

[15] Hamzavi-Zarghani Z, Yahaghi A, Matekovits L (2019). Reconfigurable metasurface lens based on graphene split ring resonators using pancharatnam-berry phase manipulation. J. Electromagn. Waves Appl..

[16] Hamzavi-Zarghani Z, Yahaghi A, Matekovits L, Farmani A (2019). Tunable mantle cloaking utilizing graphene metasurface for terahertz sensing applications. Opt. Express.

[17] Chen M (2017). Wideband tunable cross polarization converter based on a graphene metasurface with a hollow-carved “h” array. IEEE Photon. J..

[18] Chen M, Xiao X, Chang L, Wang C, Zhao D (2017). High-efficiency and multi-frequency polarization converters based on graphene metasurface with twisting double l-shaped unit structure array. Opt. Commun..

[19] Praditwong K, Harman M, Yao X (2010). Software module clustering as a multi-objective search problem. IEEE Trans. Softw. Eng..

[20] Sivanandam, S. Introduction to genetic algorithms (2008).

[21] Chen CF (2011). Controlling inelastic light scattering quantum pathways in graphene. Nature.

[22] Xiaoyong He, Feng Liu, Fangting Lin, Wangzhou Shi (2018). Graphene patterns supported terahertz tunable plasmon induced transparency. Opt. Express.

[23] Hanson GW (2008). Dyadic green’s functions and guided surface waves for a surface conductivity model of graphene. J. Appl. Phys..

[24] Hao J (2009). Optical metamaterial for polarization control. Phys. Rev. A.

